# Supplementary material for: Reliability and Convergent Validity of Endurance Indices Derived from Near-Infrared Spectroscopy and Electromyography during a Bilateral Hanging Task in Amateur Rock Climbers
Source: J Funct Morphol Kinesiol. 2024 Sep 10;9(3):161. doi: 10.3390/jfmk9030161 (PMC11417833; doi:10.3390/jfmk9030161)
Supplement: Supplementary file 1 [file jfmk-09-00161-s001.zip › jfmk-3139600-supplementary.pdf]

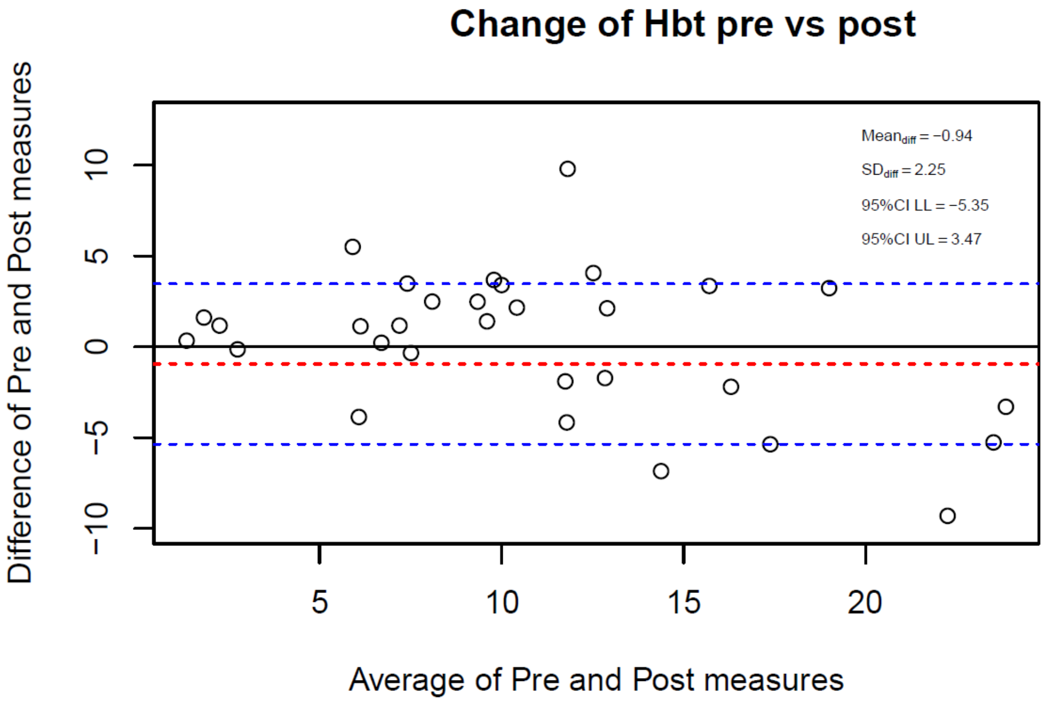

Figure S1. Bland–Altman plot for the difference between the concentrations of oxyhemoglobin and deoxyhemoglobin.

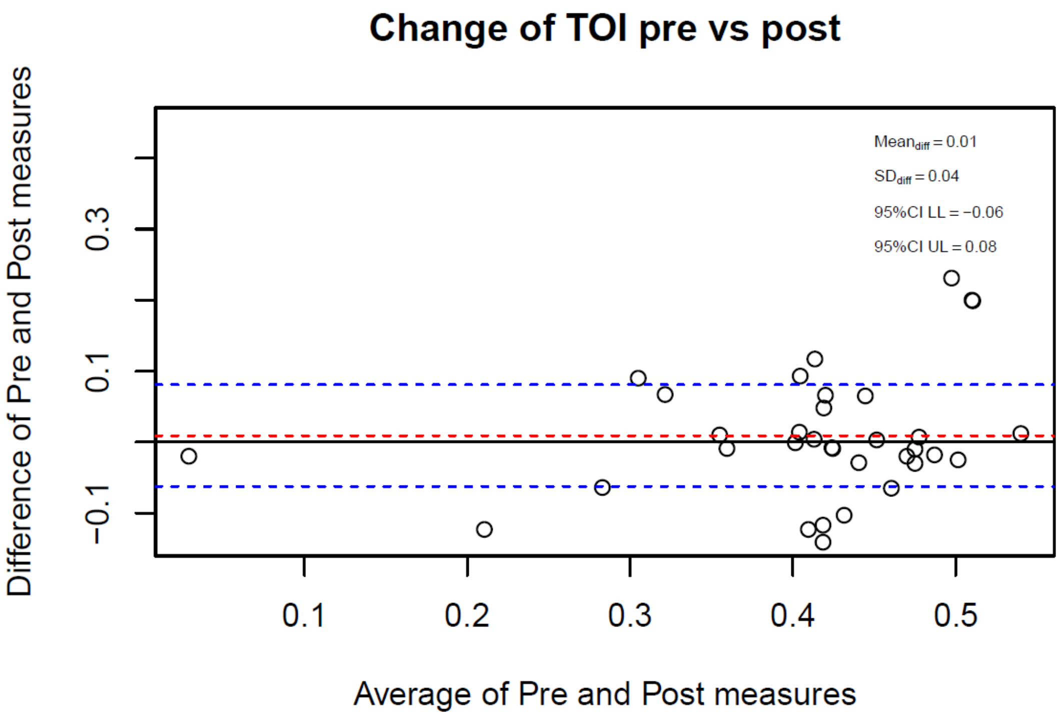

Figure S2. Bland–Altman plot for the difference between the initial and final tissue oxygenation index values.

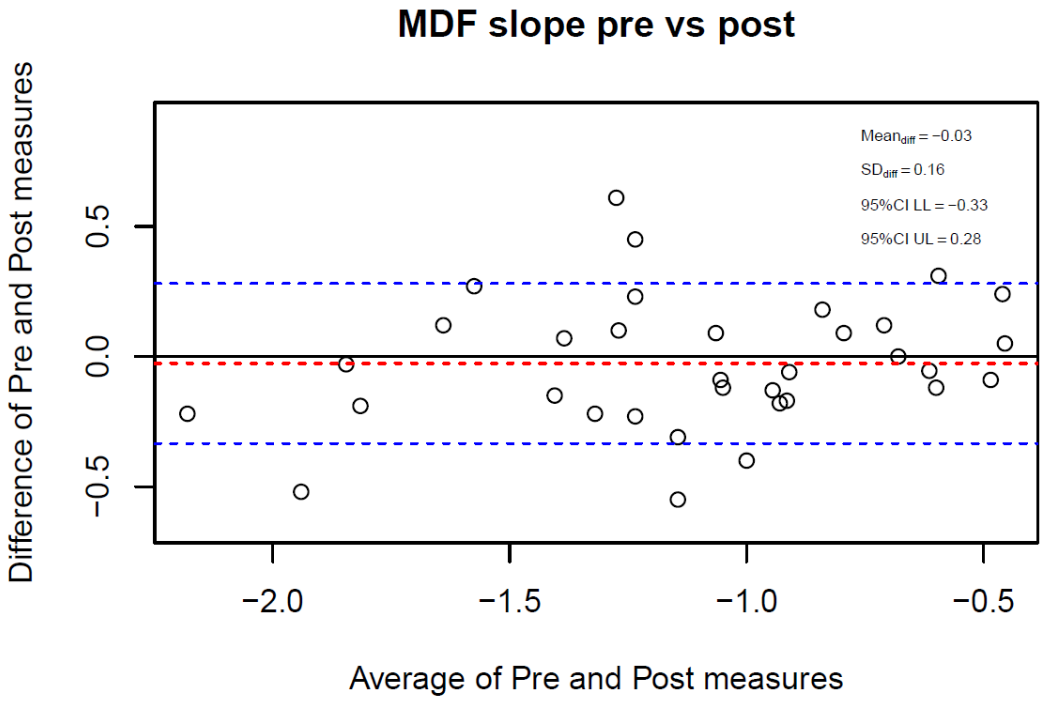

Figure S3. Bland–Altman plot for the slope of the changes in the median frequency.

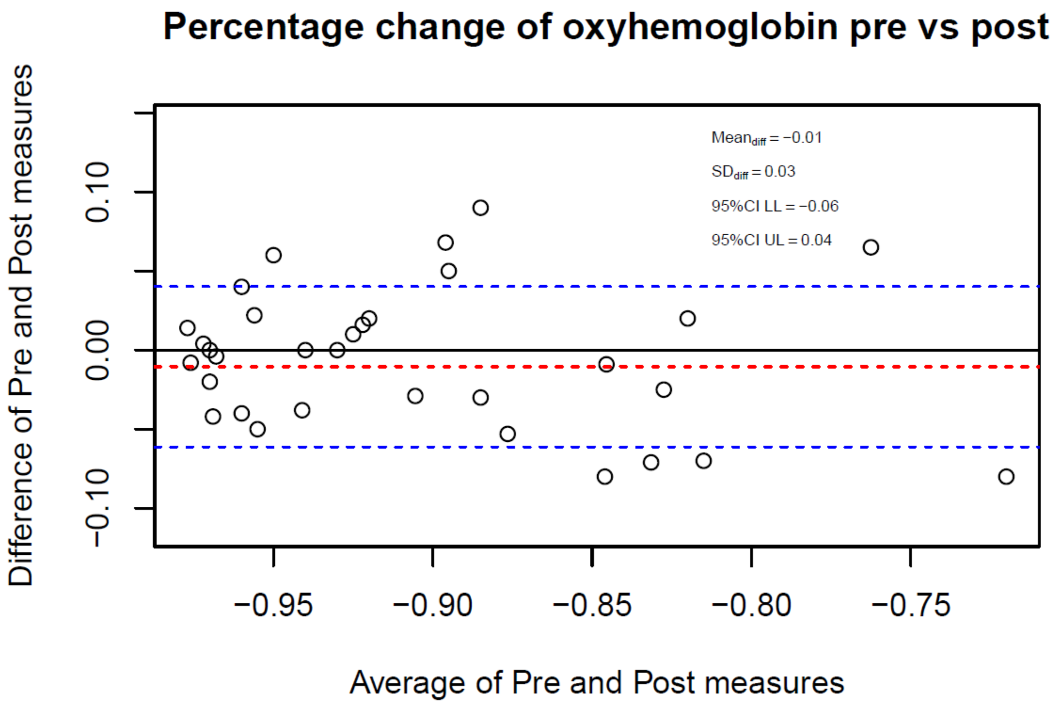

Figure S4. Bland–Altman plot for the percentage change in oxyhemoglobin concentration.

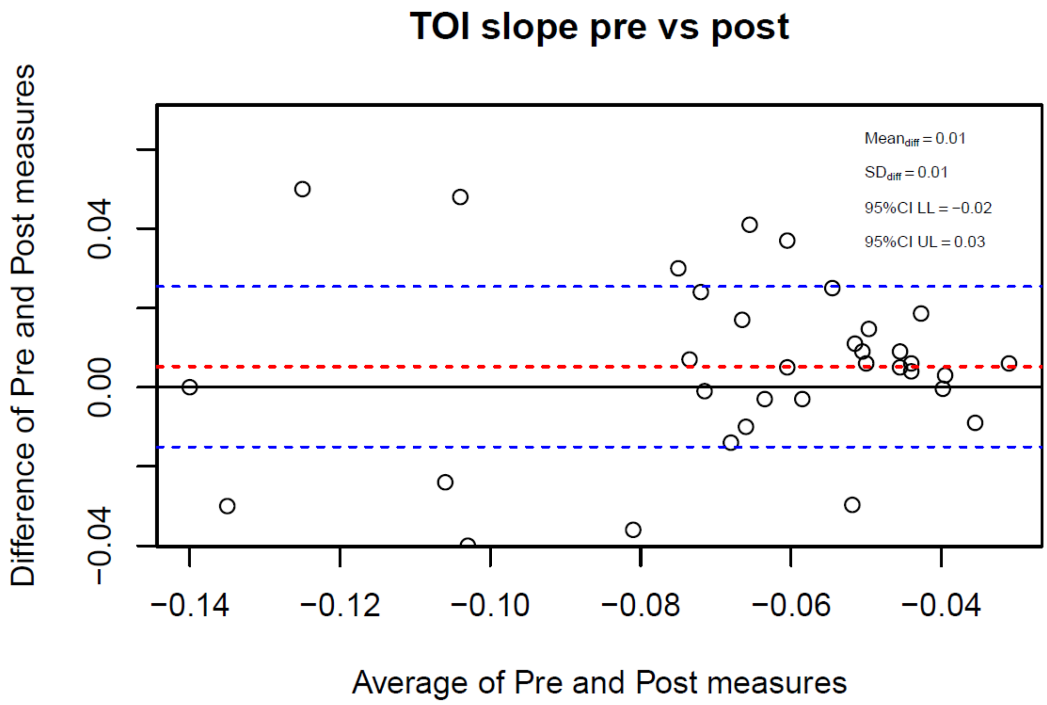

Figure S5. Bland–Altman plot for the slope of tissue oxygenation index.
